# Functional Characterization of *POLE1* Variant Fibroblasts Reveals Replication Stress and Increased Sensitivity to Genotoxic Stress

**DOI:** 10.3390/diseases14030092

**Published:** 2026-03-04

**Authors:** Enas Khdeda, Nora Naumann-Bartsch, Nawres Khdeda, Giulia Cramer, Laura S. Hildebrand, Paula Schiller, Paul Julian Wagner, Franziska Fahrmeier, Ulrike Hüffmeier, Stefanie Corradini, Luitpold V. Distel, Lukas C. F. Kuhlmann

**Affiliations:** 1 Department of Radiation Oncology, Universitätsklinikum Erlangen, Friedrich-Alexander-Universität Erlangen-Nürnberg (FAU), 91054 Erlangen, Germany; enas.khdeda@fau.de (E.K.); nawreskhdeda2@gmail.com (N.K.); giulia.cramer@uk-erlangen.de (G.C.); laura.hildebrand@uk-erlangen.de (L.S.H.); paula.schiller@fau.de (P.S.); stefanie.corradini@uk-erlangen.de (S.C.); lukas.kuhlmann@uk-erlangen.de (L.C.F.K.); 2 Comprehensive Cancer Center Erlangen-EMN (CCC ER-EMN), 91054 Erlangen, Germany; nora.naumann@uk-erlangen.de (N.N.-B.); franziska.fahrmeier@uk-erlangen.de (F.F.); 3 Department of Pediatrics and Adolescent Medicine, Universitätsklinikum Erlangen, Friedrich-Alexander-Universität Erlangen-Nürnberg (FAU), 91054 Erlangen, Germany; 4 Institute of Physiology and Pathophysiology, Friedrich-Alexander-Universität Erlangen-Nürnberg (FAU), 91054 Erlangen, Germany; paul.j.wagner@fau.de; 5 Institute of Human Genetics, Universitätsklinikum Erlangen, Friedrich-Alexander-Universität Erlangen-Nürnberg (FAU), 91054 Erlangen, Germany; ulrike.hueffmeier@uk-erlangen.de

**Keywords:** *POLE1*, DNA polymerase epsilon, fibroblasts, radiosensitivity, replication stress, homologous recombination, γH2AX, RAD51, cisplatin

## Abstract

Background/Objectives: DNA polymerase ε (Pol ε), encoded by *POLE1*, plays a pivotal role in high-fidelity DNA replication and in coordinating DNA repair. While pathogenic exonuclease-domain variants are well established in cancer, biallelic *POLE1* variants remain largely unexplored in non-malignant human cells. Methods: Here, we analyzed primary fibroblasts derived from a skin biopsy of a compound-heterozygous patient carrying two *POLE1* variants. Western blot analysis confirmed detectable Pol ε protein levels, indicating preserved protein expression despite the underlying variants. Results: Nevertheless, functional alterations were observed across multiple independent assays. Compared with healthy control fibroblasts, this patient-derived Pol ε fibroblast line exhibited reduced clonogenic survival following ionizing radiation. Surviving fractions were consistently lower across radiation doses from 2 to 4 Gy, with an approximately twofold reduction at 2 Gy and progressively greater differences at higher doses. The isoeffect dose corresponding to 10% survival was reduced relative to pooled control fibroblasts. In addition, chromosomal breakage was increased, supporting altered processing of radiation-induced DNA damage in this cellular model. Live-cell imaging and senescence assays revealed delayed proliferation and an increased proportion of senescent or senescence-like cells under baseline and genotoxic stress conditions, including enhanced senescence-associated β-galactosidase activity. Flow-cytometric analysis demonstrated S phase accumulation and G2/M arrest, consistent with replication stress and cell-cycle perturbation. Immunofluorescence staining revealed increased γH2AX foci, consistent with persistent DNA double strand breaks. RAD51 foci formation was not reduced; instead, increased RAD51 recruitment was observed under combined cisplatin and irradiation treatment, arguing against a primary defect in RAD51-mediated homologous recombination. *POLE1*-variant fibroblasts also showed impaired proliferative recovery, reduced wound closure, increased γH2AX accumulation following cisplatin exposure, suggesting heightened susceptibility to DNA crosslinking stress. Conclusions: Collectively, these findings provide the first functional characterization of a patient-derived *POLE1*-variant fibroblast cell line and indicate that altered Pol ε function may influence cellular responses to genotoxic stress. While based on primary fibroblasts from a single compound-heterozygous patient, validation in additional patient-derived or isogenic models will be required to determine the broader relevance of these findings.

## 1. Introduction

Faithful duplication of the genome during each cell cycle is essential to maintain genomic stability. In eukaryotes, three highly conserved DNA polymerases—α, δ, and ε—cooperate at the replication fork to ensure accurate DNA synthesis [1,2]. Among these, Pol ε functions as the primary enzyme for leading-strand replication [1,2]. Pol ε is a multi-subunit complex, and its catalytic subunit, encoded by the *POLE1* gene, harbors both polymerase and proofreading exonuclease activity [1]. Through its proofreading capacity, Pol ε is crucial for replication fidelity, preventing the accumulation of errors that could otherwise compromise genome integrity [2,3]. Knockout studies demonstrate that complete loss of Pol ε function is incompatible with viability in multicellular organisms, underscoring its essential role in DNA replication [2].

Beyond its role in replication, variants affecting *POLE1* have also been implicated in human disease [4]. Biallelic loss-of-function variants cause a clinical phenotype overlapping with IMAGe syndrome, often accompanied by growth restriction, adrenal and skeletal anomalies, distinctive craniofacial features, and variable immune dysfunction [4]. In addition, earlier reports describe a related presentation termed FILS syndrome—characterized by facial dysmorphism, immunodeficiency, livedo, and short stature—highlighting that biallelic *POLE1* deficiency encompasses a spectrum of developmental abnormalities rather than a single discrete entity [5]. A further case report described a patient with biallelic Pol ε-deficiency showing features reminiscent of chromosomal instability and DNA-breakage syndromes, providing additional evidence for impaired genome maintenance in this setting [6]. In contrast, exonuclease-domain missense mutations selectively impair proofreading while preserving polymerase activity, leading to an ultramutator phenotype. While exonuclease-domain mutations selectively impair the proofreading activity of Pol ε but preserve its polymerase function, thereby driving a characteristic ultramutator phenotype, biallelic loss-of-function variants are predicted to compromise the catalytic activity or stability of the polymerase complex itself. Consequently, loss-of-function variants are associated with replication stress, genome instability, and developmental phenotypes rather than hypermutation-driven tumorigenesis [4,5,6]. While such variants highlight the developmental importance of *POLE1*, missense mutations in the exonuclease domain are of particular relevance in oncology. By abolishing proofreading activity, these alterations drive a characteristic ultramutator phenotype with markedly increased base substitution rates and very high tumor mutational burden, particularly in endometrial carcinoma [7,8]. Despite their hypermutated nature, such tumors are often associated with a favorable prognosis [7]. In the germline, *POLE1* variants underlie polymerase proofreading–associated polyposis (PPAP), a hereditary cancer syndrome marked by multiple colorectal adenomas and a markedly elevated lifetime risk of colorectal cancer [8].

Collectively, these findings establish *POLE1* variants as a cause of developmental syndromes [4] as well as cancer predisposition through impaired DNA replication fidelity [7,8]. Importantly, their high neoantigen load—resulting from the ultramutator phenotype caused by proofreading-domain mutations—makes these tumors especially responsive to immune checkpoint blockade, and recent studies confirm favorable outcomes with immunotherapy [9,10]. This supports their recognition as a distinct molecular subtype with prognostic and therapeutic relevance [9,10].

Despite recent advances, the impact of *POLE1* variants on cellular responses to conventional genotoxic therapies remains poorly defined. Importantly, the functional consequences of individual *POLE1* variants may vary depending on their molecular impact, and not all biallelic variants necessarily result in complete loss of Pol ε activity.

Ionizing radiation and cisplatin were selected as genotoxic stressors because both induce DNA lesions that are tightly linked to DNA replication and homologous recombination. Ionizing radiation generates DNA double-strand breaks, whereas cisplatin induces intra- and interstrand crosslinks that stall replication forks and require coordinated replication-coupled repair processes [1,2,3]. Given the established role of Pol ε in replication fork progression and genome maintenance [1,2,3], we hypothesized that Pol ε variants would render cells particularly vulnerable to these forms of genotoxic stress. Due to the high risk of tumors [7,8], radiotherapy may be necessary. This raises the question of whether individuals with *POLE1* variants are more sensitive to radiation. In particular, the sensitivity of *POLE1*-variant cells to ionizing radiation and DNA-crosslinking agents such as cisplatin has not been systematically investigated. To address this gap, our study examines the radiation sensitivity and cisplatin response of germline *POLE1* fibroblasts, aiming to explore potential therapeutic vulnerabilities associated with altered Pol ε function.

## 2. Materials and Methods

### 2.1. Cell Lines

Three fibroblast cell lines were used in this study: one patient-derived *POLE1*-variant fibroblast cell line and two control lines, SBLF-24 and SBLF-7, derived from healthy adult donors at the University of Erlangen. The *POLE1*-variant fibroblast line originated from a compound-heterozygous patient carrying two previously undescribed *POLE1* variants. These cell lines served to compare cellular responses to cisplatin and irradiation. The use of primary human fibroblasts, including the *POLE1*-variant fibroblast line, was approved by the Ethics Committee of the Medical Faculty of Friedrich-Alexander-Universität Erlangen-Nürnberg (21_19 B and 204_17 BC). Informed consent was obtained from all adult donors. For minor donors, consent was obtained from their legal guardians, as appropriate.

### 2.2. Cell Culture and Cisplatin Treatment

All cell lines were used at comparable passage numbers (P5–P8). Cells were cultured in Dulbecco’s Modified Eagle’s Medium (PAN-Biotech GmbH, Aidenbach, Germany) supplemented with 10% fetal bovine serum (Sigma-Aldrich, now Merck, Darmstadt, Germany) and 1% penicillin–streptomycin (Gibco, Thermo Fisher Scientific, Waltham, MA, USA). Cultures were maintained at 37 °C in a humidified atmosphere with 5% CO_2_ and passaged at approximately 80% confluence. Medium was changed twice weekly. All cell lines were routinely tested for mycoplasma contamination, with no contamination detected. Across experiments, the standard treatment conditions included untreated controls, 2 Gy irradiation, cisplatin treatment, and the combination of cisplatin + 2 Gy. Deviations from these conditions are explicitly indicated in the corresponding experimental subsections. All experiments, unless stated otherwise, were performed in at least four independent replicates for each condition and cell line. All irradiations were performed using an X-ray tube operating at 120 kV (GE, Ahrensburg, Germany) with a dose rate of 1.3 Gy/min.

Cisplatin (University Pharmacy, Erlangen, Germany) was dissolved in 0.9% NaCl to prepare a 1 mg/mL stock solution. In all experiments, cells were treated with a final concentration of 22 µM. The selected concentration is within the range commonly reported in fibroblast and epithelial cell studies [11,12] and was further supported by preliminary dose–response experiments performed in our laboratory to identify a concentration that induced measurable cellular stress without causing excessive acute cytotoxicity. Cisplatin was removed after 24 h and replaced with drug-free medium. Aliquots were protected from light, stored at +4 °C, and used within one week of preparation.

### 2.3. Live Cell Microscopy

For this experiment, the *POLE1*-variant fibroblast cell line and the control line SBLF-24 were analyzed. Due to technical limitations of the live-cell imaging setup, which restricts the number of conditions that can be recorded simultaneously over extended time periods, live-cell imaging experiments were performed using Pol ε fibroblasts and SBLF-24 as a representative control. A total of 30,000 cells were seeded into each well of a 24-well plate with 0.8 mL medium per well. Immediately after seeding, cells were monitored using a live-cell imaging system (zenCELL owl, innoME, Espelkamp, Germany), with images acquired automatically at hourly intervals over 10 days. After 24 h of incubation, cisplatin was added to the designated treatment groups, followed 3 h later by irradiation with 2 Gy or 5 Gy. Growth curves were derived from automated confluence measurements; doubling times were computed by exponential fitting in Prism (GraphPad Software, San Diego, CA, USA).

### 2.4. Westernblotting

Whole-cell protein lysates were prepared using the NE-PER Nuclear and Cytoplasmic Extraction Kit (Thermo Fisher Scientific, Waltham, MA, USA; Cat. No. 78833), which enables the separation of nuclear and cytoplasmic protein fractions. Lysates were stored at −80 °C until further processing. After thawing, protein samples were denatured in Laemmli sample buffer (Bio-Rad Laboratories, Hercules, CA, USA; Cat. No. 1610737) at 70 °C for 10 min. A total volume of 20 µL per sample was loaded onto a 4–20% precast polyacrylamide gel (Bio-Rad Laboratories, Hercules, CA, USA; Cat. No. 4568093) for electrophoretic separation. Proteins were transferred onto a PVDF membrane using Tris-glycine transfer buffer supplemented with 10% methanol and the transfer was performed overnight. Membranes were subsequently blocked for 30 min at room temperature in TBST containing 3% bovine serum albumin (BSA). Following washing in TBST, membranes were incubated overnight at 4 °C with primary antibodies diluted in blocking solution: anti β-actin (ab6276, Abcam, Cambdrigde, UK; dilution 1:5000) and N-terminal binding anti-*POLE1* (GeneTex, Irvine, CA, USA; Cat. No.GTX132100; dilution 1:2000). After thorough washing with TBST, appropriate secondary antibodies were applied for 1 h at room temperature: anti-mouse 680 (5470P, Cell Signaling Technology, Danvers, MA, USA) and anti-rabbit 800 (5151S, Cell Signaling Technology, Danvers, MA, USA), both diluted 1:20,000. Membranes were washed again in TBST and imaged using an iBright imaging system (Thermo Fisher Scientific, Waltham, MA, USA). The antibodies on the membranes were stripped using Restore PLUS Western Blot Stripping Buffer (Thermo Fisher Scientific, Waltham, MA, USA; Cat. No. 46430). The membranes were then incubated with a C-terminal binding anti-*POLE1* antibody (PA5-84367, Invitrogen Thermo Fisher Scientific, Eugene, OR, USA; dilution 1:2000) and processed accordingly.

### 2.5. Scratch Assay

To create a defined gap between two cell layers, a two-well culture insert (ibidi GmbH, Gräfelfing, Germany) was placed in each well of a 24-well plate, and 50,000 cells were seeded per well. The plate was incubated for 44–48 h until confluency was reached, after which the inserts were removed, leaving two cell populations separated by a gap. Fresh medium and cisplatin were then added. After an additional 3 h of incubation, the cells were irradiated with 2 Gy. The plate was subsequently transferred to the live-cell imaging system, which acquired images at hourly intervals until the gaps in the control wells were completely closed.

To evaluate the outcome, all four conditions (control, cisplatin, 2 Gy, cisplatin + 2 Gy) were compared. The closure time of the gap in control wells (Area control = 0 mm^2^) served as the reference. At the corresponding timepoint, the size of the remaining scratch area (Area condition = X mm^2^) was measured in the other conditions using Biomas Software (V3.0 7/2012, Erlangen, Germany). Relative to the initial gap size (Area initial = 0.6 mm^2^), the remaining scratch area was quantified and plotted.

### 2.6. Cell Death and Cell Cycle Measurement by Flow Cytometry

For this experiment, 200,000 cells per fibroblast line (*POLE1*-variant, SBLF-24, and SBLF-7) were seeded into T25 flasks. After 24 h, all cell lines were treated with cisplatin, and 3 h later irradiated with 2 Gy. Cells were harvested 48 h after cisplatin treatment. For cell cycle analysis, cells were fixed in 70% ethanol at 4 °C overnight and stained with 3 µg/mL of Hoechst 33342 dye (Invitrogen, Thermo Fisher Scientific, Eugene, OR, USA). Cell cycle distribution (G0/G1, S, and G2/M phases) was determined using Kaluza Analysis Software v2.3 (Beckman Coulter, Brea, CA, USA) based on DNA content histograms. Standardized gating templates were applied, and identical gating thresholds were used across all experimental conditions and cell lines to ensure consistent phase assignment, particularly for the S phase, which lacks sharply defined boundaries. For cell death analysis, cells were stained with a 1:1 mixture of Annexin V (Invitrogen, Thermo Fisher Scientific, Eugene, OR, USA) and 7-AAD (BD Biosciences, San Jose, CA, USA), using 10 µL of each reagent, and incubated for 30 min at 4 °C. Annexin V/7-AAD dot plots were analyzed using Kaluza Analysis Software with uniform quadrant settings applied across all samples. Measurements were performed using a CytoFLEX flow cytometer (Beckman Coulter, Brea, CA, USA), and data were analyzed with Kaluza Analysis Software v2.3 ( Beckman Coulter, Brea, CA, USA).

### 2.7. Colony Formation Assay

Clonogenic assays were performed using the *POLE1*-variant fibroblast cell line and the control lines SBLF-7 and SBLF-24. For each condition, a defined number of cells (300–20,000 per dish, depending on treatment condition and expected survival) was seeded into Petri dishes based on manual cell counts using a Neubauer hemocytometer (Marienfeld-Superior, Paul Marienfeld GmbH & Co. KG, Lauda-Königshofen, Germany). Each dish contained 3–10 mL of culture medium, adjusted to dish size. 24 h after seeding, cells were irradiated with single doses ranging from 1 to 4 Gy. At 48 h post-irradiation, the medium was replaced with fresh medium. Cultures were monitored daily under a phase-contrast microscope to determine the optimal time point for staining. After 14 days, colonies were fixed and stained with methylene blue (Carl Roth, Karlsruhe, Germany) for 45 min at room temperature. Colonies consisting of ≥50 cells were manually counted, as standard in clonogenic survival assays [12].

### 2.8. Senescence Assay

All three fibroblast cell lines—*POLE1*-variant, SBLF-24, and SBLF-7 were included in this experiment. Initially, 50,000 cells per condition were seeded into T25 flasks containing 5 mL of culture medium. After 24 h, the medium was replaced with fresh medium, immediately followed by treatment with cisplatin. Three hours later, the cells were irradiated with 2 Gy under the respective experimental conditions. Ten days post-seeding, staining and analysis were performed. For this purpose, cells were incubated in medium supplemented with 2% FBS and then transferred into FACS tubes. First, the cells were treated with Bafilomycin A1 from Streptomyces griseus (EMD Millipore, Merck KGaA, Darmstadt, Germany) and incubated for 30 min at 37 °C in a humidified atmosphere containing 5% CO_2_. Hoechst 33342 dye was then added, followed by an additional 30-min incubation under the same conditions. Next, the cells were stained with C12FDG (5-dodecanoylaminofluorescein di-β-D-galactopyranoside; Invitrogen, Thermo Fisher Scientific, Eugene, OR, USA) and incubated for 60 min at 37 °C and 5% CO_2_. Finally, staining with Annexin V and 7-AAD (1:1 ratio) was performed, and the cells were incubated for 30 min at 4 °C. Flow cytometric analysis was conducted using a CytoFLEX flow cytometer (Beckman Coulter, Brea, CA, USA), and data were analyzed using Kaluza software (Beckman Coulter, Brea, CA, USA). Senescent cells were identified based on increased C12FDG fluorescence intensity using a predefined fixed threshold. Only C12FDG-positive cells were included in the senescence analysis.

Senescence was also assessed in fibroblasts derived from the clonogenic survival assays. Colonies obtained under all treatment conditions (0, 1, 2, and 4 Gy) were examined, and approximately 1500 cells were evaluated per condition. Under normal conditions, fibroblasts display an elongated spindle-like morphology with a typical length-to-width ratio of about 3:1 to 10:1. For the purpose of this study, cells with a length-to-width ratio of ≤3:1 were classified as displaying a senescence-like morphology, reflecting the characteristic flattened and enlarged morphology. While cellular senescence involves multiple biological hallmarks, a morphology-based classification represents a widely used practical approach for assessing senescence-like morphological changes in primary fibroblasts, allowing consistent and reproducible scoring across all experimental conditions in this study.

The percentage of senescent (C12FDG-positive) or senescence-like (morphology-based) cells was calculated relative to the total number of cells analyzed.

### 2.9. Immunostaining Assay

The fibroblast cell lines Pol ε, SBLF-24, and SBLF-7 were included in this experiment. A total of 30,000 cells per well were seeded into 8-well chamber slides with 0.5 mL medium per well. After 24 h, cells were treated with cisplatin and, 3 h later, irradiated with 2 Gy. At 24 h post-treatment, cells were fixed with 4% formaldehyde in PBS (Carl Roth GmbH + Co. KG, Karlsruhe, Germany) for 15 min at room temperature. Blocking was performed overnight at 4 °C in 1% bovine serum albumin (BSA; SERVA Electrophoresis GmbH, Heidelberg, Germany). Primary antibody incubation was carried out overnight at 4 °C in 1% BSA with Ki-67 (rat, 1:1000; Invitrogen, Thermo Fisher Scientific, Waltham, MA, USA), Rad51 (rabbit, 1:200; Abcam, Cambridge, UK) and γH2AX (mouse, 1:200; Merck Millipore, Burlington, MA, USA). After washing, cells were incubated for 90 min at room temperature with the appropriate secondary antibodies, Donkey anti-rat Alexa Fluor 750 (1:200; Abcam, Cambridge, UK) and Donkey anti-rabbit Alexa Fluor 555 (1:200; Invitrogen, Thermo Fisher Scientific, Waltham, MA, USA), whereas γH2AX was detected using a primary antibody directly conjugated to Alexa Fluor 488. Nuclei were counterstained with Vectashield mounting medium containing DAPI (Vector Laboratories, Burlingame, CA, USA). Fluorescence images were acquired with an Axio Imager Z2 (Zeiss, Göttingen, Germany) and analyzed using the Metafer 4 slide-scanning system (MetaSystems, Altlussheim, Germany). For analysis, only Ki-67-positive cells were included. RAD51 foci quantification was performed in these Ki-67-positive cells, whereas γH2AX staining was used for parallel assessment of DNA damage but was not required for RAD51-specific analyses.

### 2.10. Fluorescence In Situ Hybridization (FISH) Analysis

FISH was performed to visualize specific chromosomes using fluorescently labeled probes. Fibroblasts were grown to ~80% confluence and cultured in 0.2% FBS medium for 48 h. Cells were then split and transferred into 10% FBS medium supplemented with 6 µM aphidicolin (Calbiochem, cat. no. 178273) to synchronize them in early S phase by blocking DNA replication. After overnight incubation, cells were washed with PBS and cultured in fresh 10% FBS medium containing 2% colcemid (Life Technologies GmbH, Darmstadt, Germany, Cat. No. 15210040, Germany) to arrest mitosis in metaphase. One flask of each cell line was irradiated with 2 Gy immediately after medium change.

After 24 h, mitotic cells were harvested by mitotic shake-off, and supernatants as well as PBS rinses were collected. Mitotic cells were then treated with 0.56% potassium chloride for 12 min at 37 °C to induce cell swelling, followed by fixation in methanol/acetic acid (3:1). Cell suspensions were dropped onto slides and air-dried for 24–48 h. Slides were treated with pepsin, dehydrated in ethanol series (70%, 90%, 100%), and hybridized with fluorescent probes (MetaSystems, Altlussheim, Germany, Cat. No. D-0328-200-MC) labeling chromosomes 1, 2, and 4 in red, green, and yellow, respectively. Chromosomes were counterstained with DAPI. Since chromosomes 1, 2, and 4 together represent 22% of the genome, they were selected as indicators of overall chromosomal integrity. Hybridization was performed for 48 h at 37 °C. After washing, slides were mounted with 30 µL Vectashield Plus (BIOZOL, cat. no. VEC-H-2000-10) and coverslipped.

Fluorescence images were acquired using an Axio Imager Z2 microscope (Zeiss, Germany) at 630× magnification and analyzed with Biomas Software v6.5 (Erlangen, Germany). Chromosomal aberrations (e.g., insertions, translocations) were quantified as breaks per metaphase (B/M), following an established FISH-based scoring approach [13]. For each cell line, the 0 Gy value was used as baseline and subtracted from the 2 Gy value. A B/M value of 0.4 indicates normal radiation sensitivity, whereas ≥0.5 reflects increased sensitivity.

### 2.11. Statistical Analysis

For all experiments, raw data were recorded in Microsoft Excel 2016 (Microsoft Corporation, Redmond, WA, USA), where values were independently recorded per biological replicate. Graphical representations and statistical analyses were subsequently performed using GraphPad Prism 10 (GraphPad Software, San Diego, CA, USA). Statistical significance was assessed using an unpaired, two-tailed Mann–Whitney U test. Within each cell line, predefined pairwise comparisons were performed between untreated controls and cisplatin-treated cells, as well as between irradiation alone (e.g., 2 Gy or 5 Gy, where applicable) and the corresponding combination treatment (irradiation plus cisplatin). Between cell lines, patient-derived *POLE1*-variant fibroblasts were compared with control fibroblasts under identical experimental conditions (control versus control, cisplatin versus cisplatin, irradiation versus irradiation, and combination treatment versus combination treatment). All comparisons were defined a priori and analyzed independently based on the experimental design. Statistical comparisons were performed between predefined experimental pairs as specified above. Given the hypothesis-driven design and limited number of planned pairwise comparisons, no additional adjustment for multiple testing was applied. Data are presented as mean values of these independent experiments. Exact *p*-values are reported. *p*-values < 0.05 were considered statistically significant.

Given the relatively small sample size per condition (typically *n* = 4) and the number of predefined pairwise comparisons, the statistical power to detect small effect sizes may be limited. In addition, although comparisons were defined a priori, the absence of formal correction for multiple testing may increase the risk of type I error. Therefore, findings should be interpreted in the context of the predefined hypothesis-driven design and considered exploratory where appropriate.

## 3. Results

**Figure 1:**
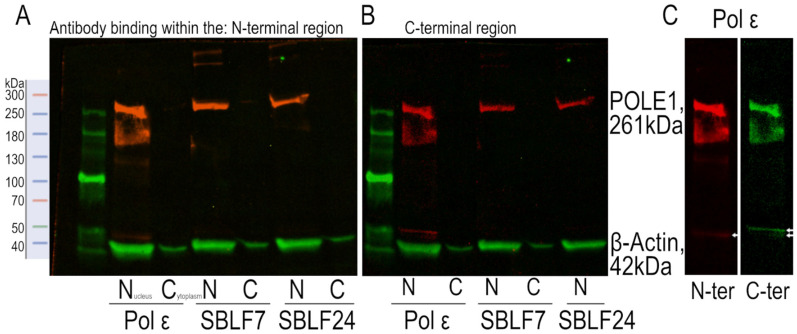
Pol ε protein in Western Blot in the *POLE1*-variant fibroblast cell line and controls. Western blot of nuclear (N) and cytoplasmic (C) proteins from the *POLE1*-variant fibroblast cell line, as well as the SBLF7 and SBLF24 cell lines. The size markers are shown on the left, with the visible light markers and the fluorescent markers in green. *POLE1* is shown in red and β-actin in green. (**A**) An N-terminal binding antibody and (**B**) a C-terminal binding antibody were used. (**C**) *POLE1* detected in comparison with the N-terminal antibody (N-ter) and with the C-terminal antibody (C-ter). The upper of the two white arrows points to a band detected only by the N-terminal antibody.

We investigated the cellular response to genotoxic stress in fibroblasts carrying biallelic *POLE1* variants. Primary fibroblasts were derived from a compound-heterozygous patient harboring two pathogenic *POLE1* variants: c.51dup (exon 1) and c.2026+3G>T (exon/intron 19). The protein was detected in Western blot analysis. The 261 kDa protein was found in the *POLE1*-variant fibroblast cell line as fragments, but also as a protein with a size corresponding to that of the protein in the control cell lines (Figure 1A–C).

Given the essential role of Pol ε in DNA replication and repair, we hypothesized that *POLE1* variants might affect cell proliferation and cellular responses to genotoxic stress. To address this, we compared the growth behavior of the *POLE1*-variant fibroblast line with that of two control fibroblast lines derived from healthy donors (SBLF-24 and SBLF-7). As an initial step, proliferation dynamics were monitored by live-cell imaging under control conditions and following treatment with cisplatin and/or ionizing radiation.

### 3.1. Reduced Proliferation Rates in Pol ε Fibroblasts

**Figure 2:**
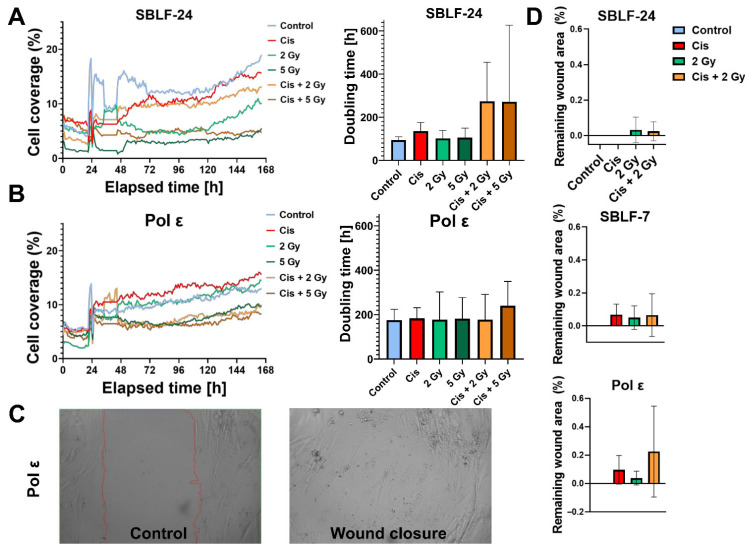
Growth behavior and migration capacity of polymerase ε (Pol ε) and control fibroblasts. (**A**) Growth curves (left) and corresponding doubling times (right) of control fibroblasts (SBLF-24) under different treatment conditions (control, Cis = cisplatin, 2 Gy, 5 Gy, Cis + 2 Gy, Cis + 5 Gy). Doubling times were calculated from exponential fits of the live-cell imaging data. (**B**) Growth curves and doubling times of Pol ε fibroblasts analyzed under the same treatment conditions. (**C**) Representative phase-contrast images of Pol ε fibroblasts in the scratch assay. The red contour marks the initial scratch area (left, control), and complete wound closure after 52 h is shown on the right. (**D**) Quantitative analysis of the remaining wound area in control fibroblasts (SBLF-24 and SBLF-7) and Pol ε fibroblasts after 52 h. Statistical significance was determined using a two-tailed Mann–Whitney U test (*p* < 0.05); values represent mean ± SD of four independent experiments (*n* = 4).

Live-cell imaging revealed that Pol ε fibroblasts proliferated more slowly than control fibroblasts under all treatment conditions. The slope of the growth curves was reduced in Pol ε cells compared with SBLF-24 controls. In SBLF-24, cisplatin treatment significantly decreased proliferation relative to untreated controls (*p* = 0.015). In contrast, Pol ε fibroblasts showed prolonged doubling times across all treatment groups without statistically significant differences between treatments. In between-line comparisons, Pol ε fibroblasts displayed longer doubling times than SBLF-24 under control conditions (*p* = 0.002) (Figure 2A,B). Representative time-lapse recordings illustrating the differential growth dynamics of Pol ε and control fibroblasts are provided in Supplementary Video S1.

### 3.2. Impaired Migration and Wound-Closure Capacity

Scratch assays demonstrated a reduced wound-closure capacity in Pol ε fibroblasts compared with control cells. While SBLF-24 and SBLF-7 fibroblasts achieved nearly complete closure under control and single-treatment conditions, Pol ε fibroblasts showed slower migration, resulting in a larger residual wound area.

Within Pol ε fibroblasts, cisplatin treatment did not reach statistical significance compared with untreated controls (*p* = 0.061). Likewise, comparison of cisplatin-treated Pol ε fibroblasts with cisplatin-treated SBLF-24 fibroblasts did not reach statistical significance (*p* = 0.061) (Figure 2D).

### 3.3. Altered Cell-Cycle Distribution in Pol ε Fibroblasts

**Figure 3:**
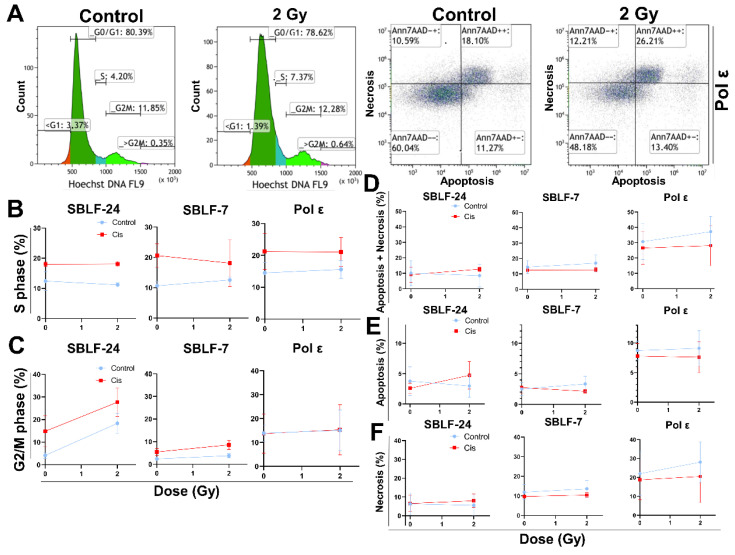
Cell-cycle distribution and cell-death analysis in polymerase ε fibroblasts (Pol ε) and control fibroblasts (SBLF-24 and SBLF-7) following cisplatin (Cis) and/or ionizing-radiation treatment. (**A**) Exemplary gating strategy of Annexin V/7-AAD staining. Ann7AAD^−−^ cells were defined as alive, Ann7AAD^+−^ as apoptotic, and Ann7AAD^++^ as necrotic. Representative flow-cytometric plots show cell-cycle distribution (left) and Annexin V/7-AAD staining profiles (right) of Pol ε fibroblasts under control conditions and after 2 Gy irradiation. (**B**,**C**) Quantitative analysis of cell-cycle profiles showing the proportion of cells in S phase (**B**) and G2/M phase (**C**) under control conditions and after treatment with cisplatin (Cis), 2 Gy irradiation, or the combination of both. (**D**,**F**) Quantification of cell death based on Annexin V/7-AAD staining. Panel (**D**) presents the combined percentage of apoptotic and necrotic cells, while panels (**E**) and (**F**) show apoptosis and necrosis separately. Blue bars represent control or irradiated conditions (2 Gy), and red bars represent treatment with cisplatin alone or in combination with 2 Gy irradiation. Statistical significance was determined using a two-tailed Mann–Whitney U test (*p* < 0.05); values represent mean ± SD of four independent experiments (*n* = 4). Cell cycle histograms and Annexin V/7-AAD dot plots were analyzed using Kaluza Analysis Software with identical gating thresholds applied across all conditions.

Flow-cytometric cell-cycle analysis revealed altered S phase distributions in Pol ε and control fibroblasts following cisplatin and/or irradiation. Cisplatin significantly increased the S phase fraction in SBLF-24 (*p* = 0.029) and Pol ε fibroblasts (*p* = 0.026) compared with their respective untreated controls. Likewise, the combination of 2 Gy irradiation and cisplatin resulted in a significantly elevated S phase fraction in SBLF-24 (*p* = 0.029) and Pol ε fibroblasts (*p* = 0.026). In SBLF-7 cells, cisplatin treatment showed a tendency toward an increased S phase fraction compared with untreated controls (*p* = 0.057). Between-line comparisons indicated a tendency toward a higher S phase fraction in Pol ε fibroblasts compared with SBLF-24 after 2 Gy irradiation (*p* = 0.057) (Figure 3B).

In the G2/M phase, significant differences were primarily observed in SBLF-7 cells. In this line, cisplatin treatment significantly increased the G2/M fraction compared with untreated controls (*p* = 0.029), and the combination of cisplatin and 2 Gy irradiation likewise resulted in a significant increase (*p* = 0.029). Between-line comparisons under controlled conditions showed significantly higher G2/M fractions in Pol ε fibroblasts compared with both SBLF-24 (*p* = 0.010) and SBLF-7 (*p* = 0.010). At 2 Gy irradiation, Pol ε fibroblasts also differed significantly from SBLF-7 (*p* = 0.010). Under combined cisplatin and 2 Gy treatment, a significant difference was observed between Pol ε and SBLF-24 fibroblasts (*p* = 0.033) (Figure 3C).

### 3.4. Enhanced Apoptotic Cell Death in Pol ε Fibroblasts

Analysis of combined apoptosis and necrosis revealed no significant treatment effects within individual cell lines. However, comparisons between Pol ε fibroblasts and the control lines revealed consistent differences. Compared with SBLF-24 fibroblasts, Pol ε cells exhibited significantly higher overall cell death under control conditions (*p* = 0.012), after cisplatin treatment (*p* = 0.012), and following 2 Gy irradiation (*p* = 0.006). Similarly, compared with SBLF-7 fibroblasts, Pol ε cells showed significantly increased overall cell death after 2 Gy irradiation (*p* = 0.006) and after combined cisplatin and 2 Gy treatment (*p* = 0.007), while control and cisplatin-treated conditions showed a tendency toward increased cell death (*p* = 0.064 each) (Figure 3D). Apoptosis and necrosis were analyzed separately to further characterize treatment-induced cell death.

In terms of apoptosis, no significant differences were observed within individual cell lines. However, Pol ε fibroblasts exhibited significantly higher apoptotic fractions compared with SBLF-24 under control (*p* = 0.012), cisplatin (*p* = 0.006), and 2 Gy irradiation (*p* = 0.012). Likewise, compared with SBLF-7 fibroblasts, apoptosis in Pol ε cells was significantly elevated under all analyzed conditions (*p* = 0.006 for each condition) (Figure 3E).

In terms of necrosis, no significant treatment effects were detected within the individual fibroblast lines. Between-line comparisons revealed significantly increased necrosis in Pol ε fibroblasts compared with SBLF-24 after 2 Gy irradiation (*p* = 0.006). Similarly, necrosis was significantly higher in Pol ε cells compared with SBLF-7 following 2 Gy irradiation (*p* = 0.024). Under control conditions and after combined cisplatin and 2 Gy treatment, comparisons between Pol ε and control fibroblasts showed tendencies toward increased necrosis (*p* = 0.067 and *p* = 0.064–0.067, respectively) (Figure 3F).

Together, these results demonstrate that Pol ε fibroblasts exhibit altered cell-cycle distribution and increased cell death compared with control lines, primarily characterized by elevated apoptosis and enhanced sensitivity to genotoxic stress. However, irradiation did not lead to a pronounced increase in early apoptotic cells. Consistent with this observation, cell cycle histograms did not show a substantial increase in hypodiploid (sub-G1) cells, indicating that extensive apoptotic DNA fragmentation was not the predominant response to irradiation. (Figure 3A–E).

### 3.5. Markedly Reduced Clonogenic Survival After Ionizing Radiation

**Figure 4:**
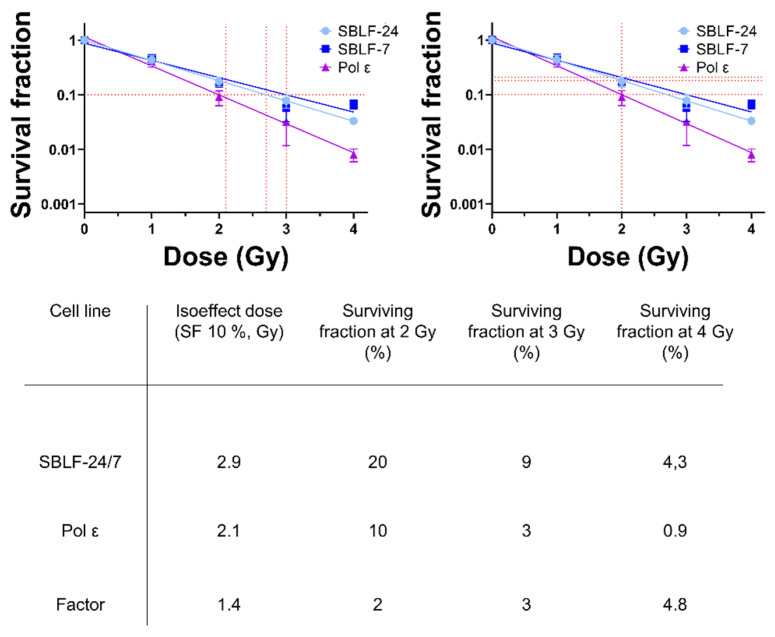
Clonogenic survival of SBLF-24, SBLF-7, and Pol ε fibroblasts following irradiation. The left panel indicates the isoeffect dose corresponding to a surviving fraction (SF) of 10%, while the right panel exemplifies survival at 2 Gy (SF2). Equivalent analyses were performed for 3 Gy and 4 Gy. The table (below) summarizes surviving fractions and sensitivity factors, calculated by comparing Pol ε with the mean of the control fibroblasts.

Next, we examined clonogenic cell death, as this assay reflects the cumulative impact of DNA damage on long-term proliferative capacity. Clonogenic survival analysis showed that the patient-derived Pol ε fibroblasts exhibited consistently lower survival fractions following ionizing radiation compared with the control cell lines SBLF-24 and SBLF-7. Across all tested radiation doses, survival of Pol ε cells was reduced relative to pooled control fibroblasts. At 2 Gy, the surviving fraction of Pol ε cells was approximately half that observed in controls, and this relative difference increased at higher doses. At 4 Gy, the sensitivity factor reached approximately 4.8, indicating a progressively greater reduction in clonogenic survival with increasing radiation dose. Consistent with these findings, the isoeffect dose corresponding to a surviving fraction of 10% (SF10) was reduced from 2.9 Gy in pooled control fibroblasts to 2.1 Gy in Pol ε fibroblasts. Together, these data indicate a dose-dependent reduction in clonogenic survival in this Pol ε fibroblast line compared with the examined control fibroblasts following ionizing radiation (Figure 4).

### 3.6. Increased Senescence-Associated Features in Pol ε Fibroblasts

To further assess stress-associated cellular phenotypes, senescence was analyzed using both flow-cytometric detection of senescence-associated β-galactosidase activity (C12FDG assay) and morphology-based classification.

**Figure 5:**
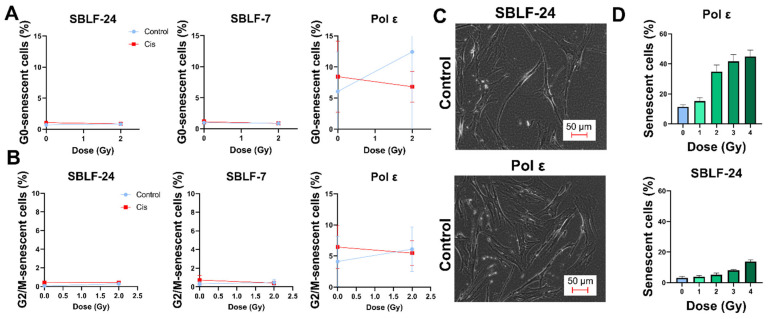
Senescence-associated changes in polymerase ε (Pol ε) and control fibroblasts following irradiation and cisplatin treatment. (**A**,**B**) Flow-cytometric quantification of senescence-associated β-galactosidase activity (C12FDG assay) in control fibroblasts (SBLF-24 and SBLF-7) and Pol ε fibroblasts. Senescent cells were identified based on increased C12FDG fluorescence intensity using a predefined fixed threshold. (**A**) Percentage of C12FDG-positive cells in the G0/G1 phase. (**B**) Percentage of C12FDG-positive cells in the G2/M phase. Blue symbols represent untreated controls, and red symbols represent cisplatin-treated conditions. Data are shown for 0 and 2 Gy irradiation as indicated. (**C**) Representative phase-contrast images of SBLF-24 and Pol ε fibroblasts under control conditions, illustrating differences in cellular morphology. Scale bar: 50 μm. (**D**) Quantification of senescence-like morphology in Pol ε and SBLF-24 fibroblasts following irradiation (0–4 Gy). Approximately 1500 cells per condition were evaluated. Cells with a length-to-width ratio ≤ 3:1 were classified as exhibiting a senescence-like morphology. For morphology-based analysis, approximately 1500 cells per condition were scored manually by two blinded investigators. For C12FDG-based analyses, data represent mean ± SD from four independent biological replicates (*n* = 4). Statistical significance was determined using an unpaired, two-tailed Mann–Whitney U test (*p* < 0.05).

Flow-cytometric quantification of C12FDG-positive cells in the G0/G1 phase did not reveal significant differences within individual cell lines between untreated controls and treated conditions. However, between-line comparisons demonstrated significantly higher proportions of C12FDG-positive cells in Pol ε fibroblasts compared with both SBLF-24 and SBLF-7 under all analyzed conditions (control, cisplatin, 2 Gy irradiation, and combined treatment; *p* = 0.029) (Figure 5A). A similar pattern was observed in the G2/M phase, where Pol ε fibroblasts exhibited significantly higher fractions of C12FDG-positive cells compared with SBLF-24 and SBLF-7 under the directly compared treatment conditions (*p* = 0.029) (Figure 5B).

Senescence-like morphology was additionally assessed following irradiation (0–4 Gy). In Pol ε fibroblasts, irradiation with 4 Gy resulted in a significant increase in cells displaying a senescence-like morphology compared with untreated controls (*p* = 0.008). A comparable increase was observed in SBLF-24 fibroblasts at 4 Gy relative to 0 Gy (*p* = 0.008). Between cell-line comparisons revealed significantly higher proportions of senescence-like cells in Pol ε fibroblasts compared with SBLF-24 under all directly compared radiation doses (0–4 Gy; *p* = 0.008) (Figure 5D).

### 3.7. Altered DNA Damage Response in Pol ε Fibroblasts

**Figure 6:**
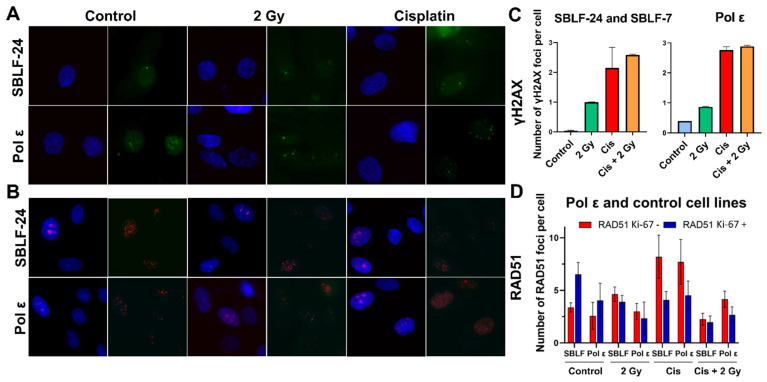
Immunofluorescence analysis of γH2AX and RAD51 foci formation in polymerase ε (Pol ε) and control fibroblasts (SBLF-24 and SBLF-7) following cisplatin (Cis) and/or irradiation treatment. Images show representative staining patterns obtained after antibody labeling and imaging. (**A**,**B**) Representative immunofluorescence images of control fibroblasts (SBLF-24) and Pol ε fibroblasts stained for (**A**) γH2AX and (**B**) Ki67 and RAD51. Nuclei were counterstained with DAPI (blue). γH2AX foci (green) and RAD51 foci (red) mark sites of DNA double-strand breaks and homologous recombination, respectively. In panel B, the left images show DAPI-stained nuclei (blue) with Ki67 positive nuclei visualized in pink, indicating proliferating cells. The corresponding right images show RAD51 foci visualized as red focal signals. For each treatment condition (control, 2 Gy irradiation, cisplatin, and the combination of cisplatin + 2 Gy), representative nuclei are shown with corresponding foci. (**C**,**D**) Quantification of γH2AX (**C**) and RAD51 (**D**) foci per cell. Data for SBLF-24 and SBLF-7 fibroblasts are pooled and referred to as “SBLF”, while Pol ε fibroblasts are shown separately. In panel D, RAD51 foci are shown separately for Ki-67-negative cells (red bars) and Ki-67-positive cells (blue bars). For RAD51 analysis, only Ki-67-positive cells were included, representing proliferating cells. Ki-67– shown for completeness. Bars represent mean ± SD from four independent biological replicates (*n* = 4). Statistical significance was determined using a two-tailed Mann–Whitney U test (*p* < 0.05).

Given the pronounced effects observed in the Pol ε cell line, we investigated whether impaired DNA double-strand break repair or altered homologous recombination contributed to this phenotype. Quantification of γH2AX foci revealed increased residual DNA damage 24 h after treatment with cisplatin and irradiation in both control and Pol ε fibroblasts. In control fibroblasts (SBLF-24 and SBLF-7, pooled), γH2AX foci were significantly elevated after cisplatin treatment compared with untreated controls (*p* = 0.008), and a significant difference was also observed between 2 Gy irradiation alone and the combined cisplatin + 2 Gy treatment (*p* = 0.008). Similarly, in Pol ε fibroblasts, γH2AX foci were significantly increased after cisplatin compared with untreated controls (*p* = 0.029), and the combination of cisplatin + 2 Gy resulted in significantly higher foci counts compared with 2 Gy alone (*p* = 0.029). Between-line comparisons demonstrated significantly higher γH2AX foci in Pol ε fibroblasts compared with pooled control fibroblasts under control conditions (*p* = 0.016), after cisplatin treatment (*p* = 0.016), after 2 Gy irradiation (*p* = 0.008), and after combined cisplatin + 2 Gy treatment (*p* = 0.008) (Figure 6C).

RAD51 foci were quantified exclusively in Ki67-positive cells; Ki67-negative cells are shown for completeness but were not included in statistical analyses. In pooled control fibroblasts (SBLF-24 and SBLF-7), RAD51 foci were significantly increased after cisplatin treatment compared with untreated controls (*p* = 0.029). In Pol ε fibroblasts, RAD51 foci were likewise significantly increased after cisplatin treatment compared with untreated controls (*p* = 0.029). Between-line comparisons revealed a trend toward higher RAD51 foci in Pol ε fibroblasts compared with pooled control fibroblasts after cisplatin treatment (*p* = 0.057). Under combined cisplatin and 2 Gy irradiation, RAD51 foci were significantly higher in Pol ε fibroblasts than in pooled control fibroblasts (*p* = 0.029) (Figure 6D).

### 3.8. Increased Chromosomal Breakage and Radiosensitivity Detected by FISH

**Figure 7:**
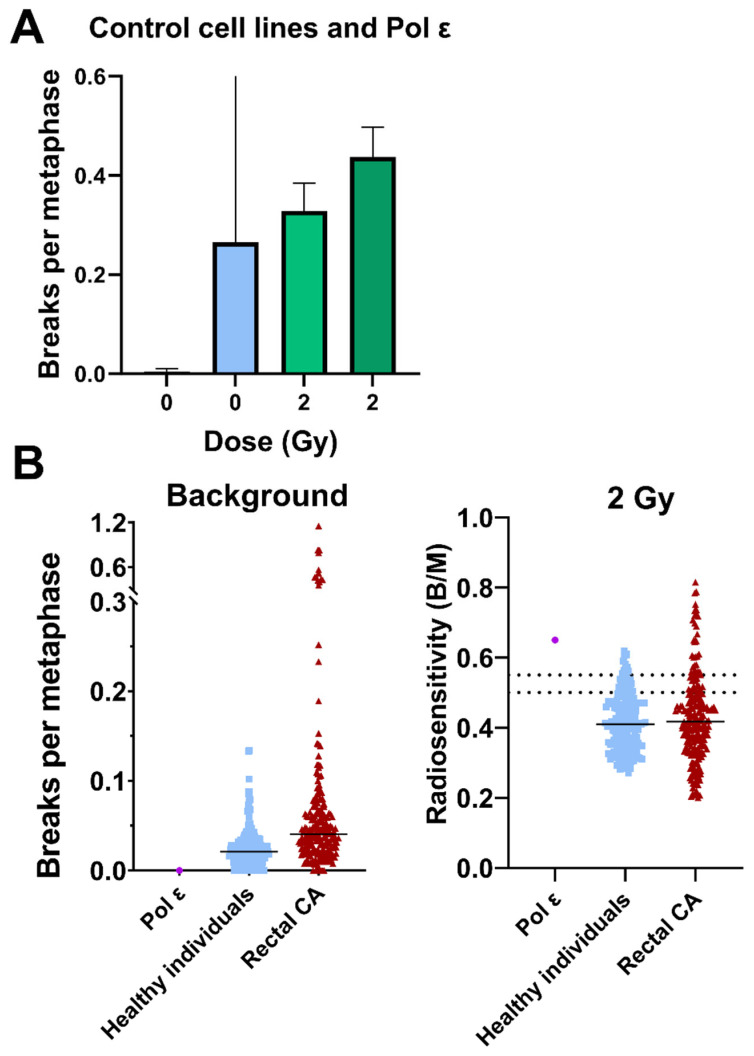
Fluorescence in situ hybridization (FISH) analysis of radiation-induced chromosomal breaks in fibroblasts and peripheral blood lymphocytes. (**A**) Metaphase FISH was performed in polymerase ε (Pol ε) fibroblasts and control cell lines (SBLF-24 and SBLF-7) to quantify chromosomal breaks per metaphase cell under control and 2 Gy irradiation conditions. For better comparability, control fibroblasts and Pol ε fibroblasts are displayed within the same graph; on the x-axis, the left bars represent the control cell lines and the right bars represent the Pol ε fibroblasts for each dose (0 Gy and 2 Gy). The values for 2 Gy irradiation are background-corrected, i.e., the 0 Gy control values were subtracted from the corresponding irradiated condition. Data represent the mean ± SD from two independent experiments (*n* = 2). (**B**) Peripheral blood FISH analysis comparing Pol ε lymphocytes with healthy individuals and rectal carcinoma patients. The left panel shows the background (0 Gy) and the right panel the radiosensitivity index (2 Gy). Dashed lines indicate established clinical reference thresholds: values ≥ 0.50 breaks per metaphase cell denote increased radiosensitivity, and values ≥ 0.55 have been proposed in this assay context as a threshold associated with increased risk of normal-tissue radiosensitivity and have been discussed in relation to potential dose-adaptation strategies. As these data derive from a single patient-derived fibroblast line (without an isogenic rescue), they do not allow definitive conclusions regarding inter-individual radiosensitivity or clinical dose adaptation.

Similar to the colony formation test, chromosome analysis for aberrations is a late endpoint of DNA damage processing and is suitable for predicting radiation sensitivity. Therefore, we performed chromosome analyses using 3-color FISH on blood and fibroblasts. FISH analysis in fibroblast cultures revealed a higher number of chromosomal breaks in Pol ε fibroblasts compared with the control cell lines under both untreated and irradiated conditions. While no breaks were detected in the control fibroblasts at baseline, Pol ε cells exhibited a clearly elevated background level of chromosomal damage. After 2 Gy irradiation, the number of breaks increased in both groups, with Pol ε fibroblasts again showing higher values than the controls (Figure 7A).

In the blood-based FISH assay, background breakage in the Pol ε lymphocytes was lower than in the healthy control cohort. Following irradiation with 2 Gy, the Pol ε sample displayed a radiosensitivity index of ~0.65 breaks per metaphase cell. This value is above the ≥0.50 threshold commonly used in this assay to classify increased chromosomal radiosensitivity; the ≥0.55 threshold has been proposed in this assay context and is shown here for reference (Figure 7B).

## 4. Discussion

This study investigated the response of *POLE1*-variant fibroblasts to genotoxic stress induced by ionizing radiation and cisplatin. We analyzed primary fibroblasts derived from a compound-heterozygous patient carrying two previously undescribed *POLE1* variants (c.51dup in exon 1 and c.2026 + 3G>T in intron/exon 19). Neither variant is listed in major clinical databases such as ClinVar, gnomAD, or LOVD (accessed February 2026). In contrast to the well-characterized exonuclease-domain mutations (e.g., P286R and V411L) that drive ultramutated endometrial and colorectal cancers [14,15], constitutional *POLE1* variants are extremely uncommon. Only isolated cases with truncating or splice-site mutations have been reported, none of which were linked to cancer predisposition [5,16,17]. Clinical features overlapping with DNA breakage and instability syndromes have been described in a patient with Pol ε-deficiency caused by a splice variant, c.4444+3A>G [6]. The c.51dup variant is predicted to induce a frameshift near the N-terminus of the Pol ε catalytic subunit, which may result in a truncated protein or altered protein stability, potentially subject to nonsense-mediated decay [5,16,17]. The c.2026+3G>T alteration affects the donor splice site downstream of exon 19 and is predicted to influence splicing, potentially altering the open reading frame and modifying Pol ε–associated cellular function [16,17]. Importantly, in our model, Western blot analysis demonstrated detectable Pol ε protein levels, indicating preserved protein expression despite the presence of these variants. Thus, the observed phenotype cannot be attributed to complete absence of Pol ε but rather suggests altered Pol ε–associated cellular function.

Given the essential role of Pol ε in replication-fork progression and high-fidelity DNA synthesis, these variants are expected to compromise genome stability and DNA repair, rendering cells more vulnerable to genotoxic stress [5,16,17]. Accordingly, this study provides new functional insight into the cellular consequences of *POLE1* variants in a patient-derived fibroblast model and its potential relevance to radiation and chemotherapy sensitivity.

In clonogenic and cytogenetic assays, *POLE1*-variant fibroblasts exhibited increased sensitivity to ionizing radiation compared with the examined control cell lines. In clonogenic survival assays, colony formation declined progressively with increasing dose, corresponding to approximately twofold lower survival at 2 Gy and nearly fivefold lower survival at 4 Gy relative to pooled controls, consistent with the reduced isoeffect dose (SF10) observed in this model. This dose-dependent reduction in clonogenic survival suggests altered processing of radiation-induced DNA lesions in this cellular context. FISH corroborated these findings at the chromosomal level, as these *POLE1*-variant fibroblasts exhibited elevated chromosomal breaks both at baseline and after irradiation. Peripheral blood lymphocytes from the same patient showed an average of 0.65 breaks per metaphase cell—above the clinical threshold of 0.50 for increased radiosensitivity [18]. Collectively, these results indicate increased radiation-associated chromosomal alterations in this patient-derived cellular context, consistent with replication stress–associated genome instability as described in related Pol ε models [19,20,21].

Notably, this contrasts with previous data from mouse embryonic stem cells carrying *POLE1* proofreading-domain mutations, where no generalized increase in radiosensitivity or chemosensitivity was observed [19]. These discrepancies likely reflect proofreading-domain hypomorphism versus catalytic loss-of-function in primary human cells, as well as differences in genetic background and DNA-damage response capacity.

To further characterize the cellular consequences of *POLE1*-variant fibroblasts, we analyzed proliferation dynamics and the induction of a senescence-like phenotype following genotoxic stress. *POLE1*-variant fibroblasts exhibited a significantly higher proportion of cells displaying senescence-associated β-galactosidase activity (C12FDG-positive cells) as well as an increased fraction of cells with senescence-like morphology compared with controls. Morphologically, these cells showed enlarged and flattened cell bodies, defined by a length-to-width ratio ≤ 3:1, which was predefined by the authors as a morphological criterion indicative of a senescence-like state [22]. Although morphology-based classification represents a widely used practical approach in primary fibroblasts, it does not by itself establish definitive cellular senescence. Therefore, we complemented morphological assessment with functional detection of senescence-associated β-galactosidase activity. The morphology-based findings should thus be interpreted with appropriate caution and in the context of the additional functional assay performed in this study.

Live-cell imaging revealed consistently slower proliferation under all conditions, with prolonged doubling times and reduced wound closure. Cisplatin treatment decreased proliferation, but combining cisplatin and irradiation did not further enhance this effect, suggesting a convergent cellular stress response. Together, these results indicate that *POLE1*-variant fibroblasts are prone to radiation- and drug-induced senescence-like phenotypic changes, accompanied by reduced proliferative capacity. This phenotype is consistent with the established role of Pol ε in maintaining replication-fork stability and limiting replication-stress–associated DNA damage [23].

Flow-cytometric profiling revealed an increased proportion of *POLE1*-variant cells in S phase under both baseline and irradiated conditions, consistent with replication stress and delayed S phase progression. Accumulation in G2/M after irradiation and cisplatin treatment is consistent with engagement of cell-cycle checkpoints in response to unresolved replication-associated damage and/or DNA lesions; however, because we did not directly measure checkpoint signaling markers, we interpret this as an indirect readout rather than definitive proof of checkpoint activation. Regarding cell death, *POLE1*-variant fibroblasts exhibited increased apoptotic fractions compared with controls, whereas necrosis remained limited. This pattern is consistent with an increased apoptotic response in the context of replication-associated DNA damage [24,25]. However, the overall magnitude of apoptosis remained limited, consistent with the well-described tendency of human fibroblasts to preferentially undergo senescence rather than acute apoptosis following genotoxic stress [24,25]. Importantly, the increased radiosensitivity observed in *POLE1*-variant fibroblasts is therefore not primarily driven by acute apoptosis, but rather reflects impaired DNA repair capacity, persistent replication-associated damage, and reduced clonogenic survival.

To explore the mechanistic basis of this phenotype, we examined RAD51 and γH2AX foci formation. RAD51 foci were quantified exclusively in Ki67 positive cells to account for the cell-cycle dependence of RAD51 recruitment. Under these cell-cycle–controlled conditions, RAD51 foci formation was not reduced in *POLE1*-variant fibroblasts; instead, increased RAD51 recruitment was observed under combined cisplatin and irradiation treatment. Because RAD51 focus formation is restricted to S/G2 phases, altered cell-cycle distribution represents an important consideration when interpreting RAD51 foci in the context of replication-associated DNA damage and homologous recombination processes [19,20,21]. Consequently, γH2AX foci accumulated under most conditions, including basal levels, reflecting unresolved DNA damage signaling (and, depending on lesion type, may also include DSB-associated γH2AX). Notably, the elevated γH2AX foci already present at baseline align with the proposed mechanism and provide a mechanistic baseline for hypersensitivity to additional exogenous genotoxic stress. The constitutive γH2AX signal in untreated *POLE1*-variant cells supports chronic replication stress, as described for Pol ε dysfunction [19,26]. Interestingly, control fibroblasts showed a more pronounced γH2AX peak at 2 Gy, consistent with more rapid initiation and/or resolution of DNA damage signaling [26,27]. Together, these findings indicate persistent replication-associated DNA damage signaling without evidence for reduced RAD51 recruitment. However, replication fork–specific mechanisms were not directly assessed in this study, and assays probing replication-fork dynamics at the molecular level (such as BrdU or RPA immunofluorescence) will be required in future work to further delineate the underlying mechanisms.

In contrast to previously reported *POLE* proofreading-domain models that showed no generalized radio- or chemosensitivity [28], our results reveal cell-type–specific vulnerabilities of primary human fibroblasts consistent with the replication-stress paradigm of Pol ε dysfunction [19,20,21]. This aligns mechanistically with recent studies demonstrating that loss of the accessory subunits POLE3 and POLE4 causes pronounced sensitivity to PARP inhibitors and, in certain contexts, to ATR inhibitors, independent of canonical HR defects [29,30]. Although these alterations are distinct from catalytic *POLE1* loss-of-function, they share the unifying feature of replication-gap–driven fragility, providing a mechanistic rationale for the platinum and radiation susceptibility observed in our patient-derived fibroblast model.

A key limitation of this study is that all functional analyses were performed in a single patient-derived *POLE1*-variant fibroblast line without an isogenic rescue model. Although two independent control fibroblast lines were included for comparison, non-isogenic differences and inter-individual genetic variability cannot be fully excluded. Consequently, the observed phenotype cannot be assumed to represent all *POLE1*-variant contexts, as variant-specific effects and patient-dependent differences in DNA damage responses may exist. The findings should therefore be interpreted as reflective of this specific cellular model and considered hypothesis-generating. Validation in additional independent *POLE1*-variant cases and genetically engineered isogenic systems will be required to determine the broader generalizability and clinical relevance of these observations. From a clinical perspective, these findings suggest that *POLE1* variants may be associated with increased chromosomal radiosensitivity in specific cellular contexts. However, any implications for radiotherapy dose adaptation remain speculative and require validation in larger cohorts before clinical recommendations can be considered.

## 5. Limitations

Apoptosis was assessed using the available readouts in this study; however, complementary apoptosis markers such as caspase activation or PARP cleavage were not analyzed. Therefore, conclusions regarding apoptosis should be interpreted as hypothesis-generating and require further validation.

In addition, all experiments were conducted in a single patient-derived *POLE1*-variant fibroblast line without an isogenic rescue model. Therefore, the findings should be interpreted as hypothesis-generating and require validation in additional independent or genetically engineered models.

Replication fork–specific assays, such as BrdU incorporation or RPA immunofluorescence, were not performed in this study. Inclusion of such analyses in future work would help to further dissect the underlying molecular mechanisms associated with altered Pol ε–related cellular responses.

## 6. Conclusions

This study provides a functional characterization of primary human fibroblasts carrying biallelic *POLE1* variants and identifies altered cellular responses to genotoxic stress in this patient-derived model. Despite detectable Pol ε protein expression, the *POLE1*-variant fibroblasts exhibited reduced clonogenic survival following ionizing radiation, increased chromosomal instability, altered cell-cycle distribution, and enhanced induction of senescence-associated features.

Together, these findings support a replication stress–associated phenotype in this specific cellular context and suggest that *POLE1* variants may influence cellular responses to DNA-damaging therapies. Importantly, as all experiments were performed in a single patient-derived fibroblast line without isogenic rescue, the results should be interpreted as hypothesis-generating. Validation in additional independent *POLE1*-variant cases and genetically engineered models will be required to determine the broader mechanistic and potential clinical relevance of altered Pol ε–associated cellular responses.

## Data Availability

The data used and analyzed during the current study are available from the corresponding author on reasonable request.

## Supplementary Materials

The following supporting information can be downloaded at: https://drive.google.com/file/d/1E3mGNmQ6_V3F-D-shUOTXDrnjUYWI2_q/view?usp=sharing (accessed on 3 December 2025); Video S1: Time-Lapse Comparison of Growth Dynamics in Pol ε and Control Fibroblasts.

## Abbreviations

The following abbreviations are used in this manuscript:

FISH	fluorescence in situ hybridization
IMAGe	intrauterine growth restriction, metaphyseal dysplasia, adrenal hypoplasia congenita, and genital anomalies
FILS	facial dysmorphism, immunodeficiency, livedo, and short stature
POLE1	DNA polymerase epsilon catalytic subunit 1
Pol ε	DNA polymerase epsilon
PPAP	polymerase proofreading–associated polyposis
RAD51	RAD51 recombinase
γH2AX	phosphorylated histone H2A.X
